# Jarvis’ Surgical Adjuster

**Published:** 1848-05

**Authors:** 


*Jarvis' Surgical Adjuster.*—Mr. Editor:—Your readers are doubtless acquainted with the admirable invention of Dr. Jarvis, of Connecticut, for the reduction of fractures and dislocations. This instrument, which ought to be in the possession of every surgeon, has been recently submitted to a Committee of the Army Medical Board, who have reported upon it favorably, a copy of which I forward for insertion in your valuable Journal. Having long been acquainted with the talented and ingenious inventor, I am desirous that his useful "adjuster" should meet with that favor from the profession, to which its undoubted merits entitle it.

I am very truly, yours &c.

CHARLES A. LEE, M. D.

Special Report of the Army Medical Board on Jarvis' Surgical Adjuster:

The Army Medical Board, consisting of Surgeons Finley, Stimeche, and assistant surgeon Southgate, to whom was referred the petition of Mr. Kellogg, addressed to the Hon. Secretary of

War, praying that Jarvis' Surgical Adjuster might be furnished to the Army of the United States, have the honor to report, that an examination of the instrument has satisfied them of its adaptation to the treatment of fractures and dislocations. The facility with which extension may be applied in a gentle and continuous manner, and instantly intermitted without losing the effect produced, and the ease with which the limb may be manipulated, render it an admirable resource in dislocations, which have continued unreduced from unskilfully directed efforts, to the absence and surgical aid, as well as in those which occasionally, under the best management, resist the means applied. The Board are likewise of the opinion, that it will prove an excellent means of treating fracture of the neck of the thigh bone; an accident, the treatment of which is too often followed by unsatisfactory results.

Whilst the Board take pleasure in testifying to the value of the invention, and admire it as a happy application of American ingenuity to the relief of human suffering, they are of the opinion that a very large proportion of fractures and dislocations may be satisfactorily treated by the means and appliances usually restored to; and at our military posts when well directed surgical aid is immediately rendered on the receipt of the injury, such an instrument will be seldom called for. So high an opinion, however, does the Board entertain of it, as a reliable means of relief in cases which may occasionally present themselves, that they would respectfully suggest to the Surgeon General, whether it may not be expedient to furnish the instrument to some of our large military stations, and to each divisions of our army in the field. Respectfully submitted by direction of the Board.

Signed, ROBERT SOUTHGATE,

*Assistant Surgeon, U. S. A.*

THOMAS LAWSON, *Surgeon General.* Washington, D. C.

NEW-YORK, May 25, 1848.

